# The effects of oral *Ginkgo biloba* supplementation on radiation-induced oxidative injury in the lens of rat

**DOI:** 10.4103/0973-1296.80673

**Published:** 2011

**Authors:** Seydi Okumus, Seyithan Taysi, Mustafa Orkmez, Edibe Saricicek, Elif Demir, Mustafa Adli, Behcet Al

**Affiliations:** *Department of Ophthalmology, Gaziantep University, Medical School, Gaziantep, Turkey*; 1 *Department of Biochemistry and Clinical Biochemistry, Gaziantep University, Medical School, Gaziantep, Turkey*; 2 *Department of Biochemistry and Radiation Oncology Gaziantep University, Medical School, Gaziantep, Turkey*; 3 *Department of Biochemistry and Emergency Medicine Gaziantep University, Medical School, Gaziantep, Turkey*

**Keywords:** *Ginkgo biloba* extract, antioxidant, irradiation, oxidative stress, lens

## Abstract

**Background:**

The aim of this study was to evaluate the antioxidant role of *Ginkgo biloba* (GB) against radiation-induced cataract in the rat lens after total cranial irradiation with a single 5 Gray (Gy) dose of gamma irradiation.

**Materials and Methods:**

Twenty-four Sprague-Dawley rats were used for the experiment. The rats were randomly divided into three equal groups. Group 1 did not receive GB or irradiation (control group) but received 1-ml saline orally plus sham-irradiation. Group 2 received total cranium 5 Gy of gamma irradiation as a single dose (IR group) plus 1-ml saline orally. Group 3 received total cranium irradiation plus 40 mg/kg/day GBE (IR plus GBE group). Biochemical parameters measured in murine lenses were carried out using spectrophotometric techniques.

**Results:**

Lens total (enzymatic plus non-enzymatic) superoxide scavenger activity (TSSA), non-enzymatic superoxide scavenger activity (NSSA), glutathione reductase (GRD), and glutathione-S- transferase (GST) activities significantly increased in the IR plus GBE groups when compared with the IR group. However, TSSA, GRD and GST activities were significantly lower in the IR group when compared with the control group. Lens xanthine oxidase (XO) activity in the IR group significantly increased compared to that of both the control and IR plus GBE groups.

**Conclusion:**

GBE has clear antioxidant properties and is likely to be a valuable drug for protection against gamma-irradiation and/or be used as an antioxidant against oxidative stress.

## INTRODUCTION

Ionizing radiation, such as X- and γ-rays and ultraviolet light, is known to be a cataractogenic factor in rat lenses.[1–3] Ionizing radiation is known to generate reactive oxygen species (ROS) in irradiated tissue. Because human body contains 80% water, the major radiation damage is due to the aqueous-free radicals generated by the action of radiation on water. These free radicals react with cellular macromolecules such as DNA, RNA, proteins, membrane, etc. and cause cell dysfunction and mortality. These reactions take place in tumor as well as normal cells when exposed to radiation.[1–4]

In scavenging of several ROS, including the superoxide anion radical (O_2_˙^–^), hydroxyl radical (OH˙^–^), hydrogen peroxide (H_2_O_2_) and peroxyl radicals, the *Ginkgo biloba* (GB) extract, which was prepared from the leaves of the GB tree according to a well-defined procedure, has been proposed to be prospective.[1] The Gingko tree belong to the Ginkgoaceae family. Ginkgo extract may have three effects on the human body: improvement in blood flow, protection against oxidative cell damage from free radicals and blockage of many effects of platelet-activating factor.[5] Ginkgo is also being used as a memory enhancer.[6] Although not proven by clinical studies, Ginkgo is being used without prescription for many purposes, including treatment of tinnitus, intermittent clauducation and vitiligo.[7]

To control the flux of ROS, aerobic cells have developed their own defense system, the antioxidant system, which includes enzymic and non-enzymic components. The antioxidant system consists of low-molecular weight antioxidant molecules such as glutathione (GSH) and of various antioxidant enzymes. For instance, superoxide dismutase (SOD), glutathione peroxidase (GSH-Px) and GSH. Superoxide dismutase (SOD) catalyses the dismutation of the superoxide anion radical (O_2_˙^–^)into H_2_O_2_.[8] H_2_O_2_ is generally considered a major oxidant in cataractogenesis. Addition of H_2_O_2_ to an *in vitro* system can cause oxidative damage to the lens resembling that seen in photochemical stress. *In vitro* models, the relative roles of different reactive oxygen species (ROS), such as H_2_O_2_ or the O_2_˙^–^, as well as the relative roles of the different defense mechanisms against these ROS can be studied. The major systems degrading H_2_O_2_ in the lens involve GSH-Px, glutathione reductase (GRD) and GSH.[9,10] A number of potentially toxic electrophilic xenobiotics are conjugated to the nucleophilic GSH by glutathione-S-transferases (GST) present in high amounts in cell cytosol. GST can also catalyze reactions reducing peroxides like GSH-Px.[11] Xanthine oxidase (XO) functions in purine and free radical metabolism. It also catalyzes the conversion of xanthine and hypoxanthine to uric acid and the production of O_2_˙^–^, which is potentially toxic to cellular structures.[12]

To our knowledge, there is no experimental study that simultaneously investigates the effect of GBE supplementation on total (enzymatic plus non-enzymatic) superoxide scavenger activity (TSSA), non-enzymatic superoxide scavenger activity (NSSA), GRD, GST and XO activities in the lens of rats with ionizing-induced cataracts. Therefore, in the present study, we aimed to investigate the effect of GB extract supplementation on antioxidant (TSSA, NSSA, GRD, GST) and oxidant parameters (XO) in the lens of rats with or without exposure to total cranium irradiation with a single dose of 5 Gy of gamma rays.

## MATERIALS AND METHODS

### Rats and experiments

Twenty-four Sprague-Dawley rats, 10–12 weeks old, weighing 210±15 g at the time of radiation were used for the experiment. All procedures involving the Sprague-Dawley rats adhered to the ARVO Resolution on the Use of Animals in Research. The rats were quarantined for at least 3 days before gamma irradiation, housed seven to a cage in a windowless laboratory room with automatic temperature (22±1°C) and lighting controls (12-h light/12-h dark) and fed standard laboratory chow and water *ad libitum*.

The rats were randomly divided into three equal groups. Group 1 did not receive GBE or irradiation (control group) but received 1 mL saline orally plus sham-irradiation. Group 2 received total cranium 5 Gy of gamma irradiation as a single dose (IR group) plus 1 mL saline orally. Group 3 received total cranium irradiation plus 40 mg/kg/day GBE (IR plus GBE group). The rats in the RT plus GBE group received 40 mg/kg GBE diluted in 1 mL physiological saline containing 40 mg/mL GBE extract (Tebokan Ford Drops, Abdi Ibrahim Pharmaceuticals, Istanbul, Turkey) daily orally through an orogastric tube, starting 3 days before irradiation and continuing for 7 days after irradiation (total, 10 days). One milliliter of saline was administered daily orally through an orogastric tube by starting from 3 days before irradiation and during 10 days after irradiation (total 13 days) to both the control group and the IR group.

The grading of the lenses from 0 to 4 by slit-lamp biomicroscopy (Nikon, Slit Lamp microscope NS-1, Tokyo, Japan) was made as follows: normal, clear lenses were graded as 0; lenses showing visible posterior sutures were graded as 1; lenses displaying isolated vacuoles were graded as 2; lenses with coalescing vacuoles were graded as 3; lenses with peripheral coalescing vacuoles and radial streaks extending into the central crystalline opacity were graded as 4. All the rat lenses at the beginning were graded as 0.

Prior to total cranium irradiation, the rats were anesthetized with 80 mg/kg Ketamine HCl (Ketamine Hydrocloride, Pfizer Inc., New York, NY, USA) and placed on a plexiglas tray in the prone position. While the rats in the control group received sham irradiation, the rats in the IR and the IR plus GBE groups received irradiation using a Cobalt-60 teletherapy unit (Theratron Equinox, MDS Nordion, Ottawa, Ontario, Canada) from a source-to-surface distance of 80 cm by 5 cm × 5 cm anterior fields with the total cranium gamma irradiation as a single dose of 5 Gy. The dose rate was 0.49 Gy/min. To insure that the lens received a maximal dose, a wax bolus material 0.5-cm thick was placed over the rat eyes. The central axis dose was calculated at a depth of 0.5 cm. The maximum dose is normalized to 95% on the lens.

### Biochemical analysis

Ten days after irradiation, all animals were killed by decapitation, their eyes were enucleated and the lenses were dissected immediately. Lenses were homogenized in physiological saline solution (IKA^®^ Werke GmbH and Co. KG, Staufen, Germany). The homogenate was centrifuged at 10,000 g for 1 h to remove debris. The clear upper supernatant was collected and all assays were carried out on this fraction. All the procedures were performed at +4°C.g

TSSA and NSSA assays, as indicators of tissue antioxidant capacity, were performed in the samples before and after adding trichloroacetic acid (TCA, 20%), as described.[13] First, TSSA is measured. In this method, the xanthine-xanthine oxidase complex produces superoxide radicals that react with nitroblue tetrazolium (NBT) to form a farmazone compound. TSSA activity is measured at 560 nm by detecting the inhibition of this reaction. By using a blank reaction in which all reagents are present except the supernatant sample and by determining the absorbance of the sample and blank, the TSSA activity is calculated. Second, NSSA activity is measured in TCA-treated fractions prepared by treating part of the sample with a final concentration of 20% (w/v) TCA solution (to remove all enzymes and proteins) and centrifuging at 5000×g for 30 min. After the elimination of proteins by this procedure, NSSA activity assay is performed in the supernatant fraction. GRD activity was determined by coupled spectrophotometric registration at 340 nm using GSSG as a substrate and NADPH at 37°C.[14] GST activity of the supernatant was measured by using 1-chloro-2,4-dinitrobenzene (CDNB) and GSH as described.[15] XO activity was measured spectrophotometrically by the formation of uric acid from xanthine through the increase in absorbance at 293 nm.[16] The protein content was determined by using the Bradford method.[17]

Results were expressed in U/mg protein for TSSA and NSSA and mU/mg protein for GRD, GST and XO activities. One unit of TSSA and NSSA was defined as the amount of enzyme protein causing 50% inhibition in the NBT reduction rate. Biochemical measurements were carried out using a spectrophotometer (Theratron Equinox, MDS Nordion).

### Statistical analyses

Statistical and correlation analyses were undertaken using a one-way variance analysis and Spearman’s rank correlation test, respectively. Least significant difference (LSD) multiple range test was used to compare the mean values. Acceptable significance was recorded when *P*-values were <0.05. Statistical analysis was performed with Statistical Package for the Social Sciences for Windows (SPSS, version 11.5, Chicago, IL, USA).

## RESULTS

Lens grades were determined by slit-lamp biomicroscopy. At the end of the 10th day, the lenses in the control group were classified as grade 0 and those in the RT group as grades 1 or 2. Although grade 1 cataract development was detectable in seven rats in the RT group, it was detectable in only one rat in the RT plus GBE group. Biochemical parameters and the cataract situation are shown in Tables 1 and 2, respectively. Lens TSSA, NSSA, GRD and GST activities significantly increased in the IR plus GBE groups when compared with the IR group. However, TSSA, GRD and GST activities were significantly lower in the IR group when compared with the control group. Lens XO activity in the IR group significantly increased compared with that of both the control and the IR plus GBE groups.

**Table 1:** Mean ± SD of total (enzymatic plus non-enzymatic) superoxide scavenger activity, non-enzymatic superoxide scavenger activity, glutathione reductase, glutathione-Stransferases and xanthine oxidase activities in rat lenses

	Control group	IR group	IR plus GBE group
TSSA U/mg protein	37.8 ± 6.2^b^	25.9 ± 4.9	46.2 ± 9.1^c^^d^
NSSA U/mg protein	8.3 ± 0.8	7.7 ± 0.9	10.9 ± 0.8^c^^e^
GRD mU/mg protein	55.7 ± 6.3^c^	44.9 ± 3.8	51.2 ± 4.1^a^
GST mU/mg protein	25.9 ± 7.2^c^	13.6 ± 4.9	34.8 ± 6.8^c^^d^
XO mU/mg protein	3.48 ± 0.22^b^	3.91 ± 0.24	3.56 ± 0.34^a^

a : *P* < 0.05

b : *P* < 0.01

c : *P* < 0.001 vs. irradiation group

d : *P* < 0.05

e : *P* < 0.001 vs. control group

**Table 2:** Cataract situation in the rat lenses examined by slit-lamp microscopy in the control, GBE and IR plus GBE groups

	Situation of cataract		
	Present	Absent	Total
Control group	0	8	8
IR group	7	1	8
IR plus GBE group	1	7	8
Total	8	16	24

Correlation analysis revealed a significant negative correlation between lens tissue XO and TSSA (r = -0.83, *P*< 0.01) in the IR plus GBE group. However, no correlation could be found among the parameters in the other groups.

## DISCUSSION

Against the deleterious effects of the oxidative damage caused by free radicals the GBE, which acts as an antioxidant, scavenges reactive oxygen and nitrogen radicals. It not only protects cells from alloxan- or light-induced stress and the nuclear DNA from single strand breaks by entering intact cells but also effectively inhibits chemically induced apoptosis and accelerates corneal wound healing. It also increases ocular blood flow velocity in the ophthalmic artery and protects against retinal damage and retinal ischemia-reperfusion injury. Its protecting the lens against selenite-induced lens opacification is due to its beneficial effects like antioxidant, antiapoptotic and cytoprotective properties.[1,18]

In this study, we showed that the irradiation caused a significant decrease in the activities of antioxidant enzymes and also increases in oxidant enzyme activities in rat lenses. Radiation-induced increase in XO activity, an oxidant enzyme, was prevented by GBE, which was reported for the first time in this study. In agreement with the results of our previous study, melatonin prevented radiation-induced increase in XO activity in rat lenses also.[12] Increased lipid peroxidation levels have been reported in the lens of irradiation-administered rats.[1,12,19] The possible mechanisms that irradiation increases oxidative stress include disruption of the mitochondrial respiratory chain leading to leakage from the electron transport chain in rats, depletion of cellular GSH level and decreased activities of antioxidant enzyme and increased activity of oxidant enzyme, i.e. XO, in rat lenses. XO is an important source of O2˙^–^ in cells and tissues. There is growing evidence that O_2_˙^–^ radicals produced by XO are primarily responsible for the cellular deterioration associated with several conditions.[20] It seems that O_2_˙^–^ and H_2_ O_2_ are the main sources of radiation-induced free radical production that deplete the cellular GSH level, which has a central role in the antioxidant defense in the cell. H2 O2 can interact with metal ion or O_2_˙^–^ to produce OH˙^–^, a very destructive radical. According to some studies, after irradiation accompanied by enhancement of lipid peroxidation, a marked depletion in the antioxidant system was reported. Normally, the natural defense system can protect against oxidative damage.[12,19,21]

In our study, we found a significant reduction in GRD and GST activities in the IR group and also a significant increase in GRD and GST activities in the IR plus GBE group in rat lenses. This reduction in the IR group could be due to an enhanced utilization of GSH, present at high concentration in the lens, redox cycle as an attempt to detoxify the free radicals generated by irradiation. Supplementation of GBE protects the endogenous GRD and GST depletion resulting from irradiation. The increase in GRD, GST, TSSA and NSSA activities suggests that GBE may be mediated through the modulation of the lens antioxidant system. These results suggest that GBE has a free radical-scavenging activity. One study has reported that GBE significantly lowered the radiation-induced lipid peroxidation in terms of malondialdehyde and increased antioxidant enzyme activities.[1] Antioxidants can cause an inhibition of lipid peroxidation in biomembranes. Oxidative damage is proposed to have a role in the pathogenesis of cataracts because significant increases in the levels of free radicals were reported in both the lens and the aqueous humor of cataract patients when compared with age-matched controls. The increased lens oxidation and cataract development could be due to a decrease in the antioxidant system.[22–24]

As a result, these results confirm the presence of oxidative stress in the IR group. The GBE has clear antioxidant properties and is likely to be a valuable drug for protection against gamma-irradiation and/or be used as an antioxidant against oxidative stress. By increasing the antioxidant enzyme activities and decreasing the oxidant enzyme activity, GBE prevented oxidative stress by scavenging free radicals generated by ionizing radiation in rat lenses.
